# Adverse events of methotrexate treatment in JIA

**DOI:** 10.1186/1546-0096-9-S1-P203

**Published:** 2011-09-14

**Authors:** J in ‘t Veld, NM Wulffraat, JF Swart

**Affiliations:** 1 VU University Medical Center, Department of Pediatric Infectious diseases, Immunology and Rheumatology, Amsterdam, The Netherlands; 2 Wilhelmina Children’s Hospital/UMC Utrecht, Department of Pediatric Rheumatology, Utrecht, The Netherlands

## Background

Methotrexate (MTX) which has been used for 20 years in Juvenile Idiopathic Arthritis (JIA) has now become the first-choice, second-line agent for children with polyarticular course JIA. New biologic drugs are used for MTX-failing patients and have been assessed in controlled trials for effectiveness. Safety data of these drugs are slowly and selectively emerging as post-marketing data. For better efficiency of collecting these data a large pharmacovigilance registry (PharmaChild) is being constructed supported by the European Union. This will enable the pediatric rheumatologists to overlook the whole spectrum of moderate to serious adverse events of these newer drugs and hence to advise their patients better on the effectiveness as well as the relative safety of the newer agents. The patients treated with these newer drugs need to be compared to the patients treated with MTX. To get a better understanding of all adverse events already encountered in MTX-therapy in JIA-patients we performed a meta-analysis.

## Aim

To offer insight into the incidences of the adverse events (AEs) due to treatment of JIA with MTX.

## Methods

By using the MeSH terms ‘Arthritis Juvenile Rheumatoid’ and ‘Methotrexate’, Pubmed and reference lists of selected articles were searched. Trials were included if patients fulfilled the diagnostic criteria for JIA, Juvenile Rheumatoid Arthritis or Juvenile Chronic Arthritis and when treated with MTX in doses of 2.5-40 mg/wk. Exclusion criteria were MTX used for treatment of oncologic disease or in combination with other DMARDs in the majority of patients or treatment with biologicals.

## Results

Twenty-six studies were identified. In total, 3984 patient-years of MTX-exposure in 2588 JIA-patients were analyzed. A total of 1247 adverse events were noted with an exposure-adjusted risk of 31.30% per 100 patient-years. The most common adverse events were found in the liver (8.08%, mainly elevated liver enzyme tests) and gastrointestinal events (7.23%, mainly nausea or vomiting), followed by unspecified- (6.25%), infectiologic- (2.61%), skin- (2.56%), mucosal- (1.36%), hematologic- (0.93%), neurologic- (0.90%), psychologic- (0.88%) events. Serious adverse events were seen in 0.50%. Per year of therapy MTX was temporarily discontinued in 1.26%, permanently withdrawn in 0.80% and discontinued unspecified in duration in 1.10%.

## Conclusion

In general, children with JIA tolerate MTX well. More than two-thirds of patients do not have any adverse events when treated with MTX for 1 year. The most common side effects are mild and related to elevated liver enzyme tests and nausea or vomiting. Infectiologic side effects were mentioned in only 2.61%. The risk of a serious adverse event due to MTX is 1 in every 200 patients treated for 1 year. MTX discontinuation (temporarily or permanent) due to adverse events was necessary in less than 5% per treated year.

